# Impact of Arteriovenous Access Formation on Cardiac Structure and Function in Hemodialysis Patients

**DOI:** 10.3400/avd.oa.25-00112

**Published:** 2026-04-23

**Authors:** Nadeem Ahmed Siddiqui, Mohammad Zakriya, Muhammad Anees, Farhala Baloch, Kashmala Hussain, Fareed Ahmed Shaikh, Zia Ur Rehman

**Affiliations:** 1 Section of Vascular Surgery, Department of Surgery, Aga Khan University Hospital, Karachi, Pakistan; 2 Section of Cardiology, Department of Medicine, Aga Khan University Hospital, Karachi, Pakistan; 3 Medical College, Aga Khan University, Karachi, Pakistan

**Keywords:** echocardiography, left ventricular dysfunction, major adverse cardiac event, renal dialysis, surgical arteriovenous shunt

## Abstract

**Objectives:**

We aimed to determine echocardiographic changes following arteriovenous fistula (AVF) creation and identify predictors of major adverse cardiac events (MACE) in end-stage renal disease (ESRD) patients.

**Methods:**

A retrospective study of ESRD patients who underwent AVF creation between 2014 and 2022 was conducted at a tertiary care center. Cardiac echocardiograms performed within 3 months before and at least 6 months after surgery were compared using paired t-tests. Predictors of MACE were assessed using Cox regression, with results reported as hazard ratios (HR).

**Results:**

A total of 119 patient records were included. Mean age was 58.2 ± 13.6 years, and 66 (55.5%) patients were male. Following the procedure, left atrial volume index (LAVI) increased (34.339 ± 10.361 vs. 37.964 ± 9.739, p = 0.001), whereas E′ velocity (0.052 ± 0.018 vs. 0.047 ± 0.016, p = 0.011) and left-ventricular ejection fraction (50.798 ± 11.442 vs. 47.374 ± 12.277, p <0.001) decreased significantly. Ejection fraction was maintained in 81 patients (68.07%), improved in 12 (10.08%), and declined in 26 (21.85%). Cox regression showed a significant association between preoperative atrial fibrillation (p = 0.019) and severe left ventricular dysfunction (p = 0.026) with MACE.

**Conclusion:**

AVF creation was associated with increased LAVI and decreased E′ velocity. Preoperative atrial fibrillation and severe left ventricular dysfunction were associated with increased risk of postoperative MACE.

## Introduction

Chronic kidney disease (CKD) is a progressive condition that can lead to end-stage renal disease (ESRD) if left untreated or poorly managed and has emerged as a major global problem in the last decade affecting more than 50 million patients in the United States alone.^1)^ The incidence of CKD in South Asia is reported to be approximately 6%–8%, with studies from the United Kingdom showing that South Asian individuals present with more advanced disease and at a significantly younger age—on average 5.7 years earlier than their White counterparts.^2)^ In Pakistan, Imran et al. reported CKD to be present in 25% of the asymptomatic population in an urban city.^3)^

ESRD requires renal replacement therapy, including hemodialysis (HD) or a kidney transplant. Lower costs and greater accessibility lead nearly 90% of patients to choose HD.^4)^ Advances in HD techniques have improved outcomes, making it a life-sustaining clinical treatment.^5)^ Vascular access for HD can either be created via external catheter or via a surgically created arteriovenous fistula (AVF). Since external catheters carry the risk of development of septicemia, the “Fistula First-Catheter Last Initiative” has been recommended to reduce central venous catheter use in favor of AVFs.^6)^ Despite a 6-week maturation period, AVFs provide long-term access with lower infection risks and central line-related morbidity than catheters.^7)^

AVFs shunt arterial blood to the venous system, significantly increasing cardiac preload and output. This may increase cardiac workload, resulting in structural and functional cardiac changes. Reddy et al. reported anatomical and physiological alterations in both left and right ventricles long after establishing an AVF, which can further lead to pulmonary hypertension and congestive cardiac failure.^8)^ Alkhouli et al. have proposed AVF ligation as a therapeutic consideration in case of cardiac complications.^9)^ In this context, and given that heart failure affects approximately 45% of patients on dialysis, evaluating the cardiovascular impact of AVF creation is of critical clinical importance.^10)^

While the global guidelines for vascular access are largely informed by high-income country (HIC) data, these models often fail to account for the unique epidemiology of CKD in low-and-middle-income countries (LMICs). Patients in these regions often lack access to early screening and cardioprotective interventions, resulting in a population that presents with advanced, multi-organ complications at a much younger age than seen in the West.^2)^ Our study addresses this gap by examining the cardiac impact of AVF creation in a South Asian cohort that must endure significant hemodynamic shifts while already burdened by advanced, untreated systemic disease. Cardiac function in dialysis patients is influenced by volume overload, AVF-induced hemodynamic changes, and underlying comorbidities. Various echocardiographic markers may offer insight into myocardial stress and long-term prognosis. Their roles in AVF-associated remodeling remain understudied, especially in LMIC populations. Most existing studies originate from HICs, whereas South Asian populations in LMICs tend to develop cardiovascular disease earlier and more severely, often alongside multiple comorbidities such as diabetes and hypertension.^2)^ Given the high incidence of cardiovascular complications among HD patients, understanding these effects is important. Proper patient selection is important to mitigate the risk of cardiac dysfunction and other postoperative complications. This study aims to contribute to this knowledge gap by determining the structural and functional cardiac changes after creating AVF by studying and comparing cardiac echocardiograms and identifying predictors of major adverse cardiac events (MACE) after creation of AVF in ESRD patients. By investigating this, we aim to inform risk stratification strategies for patients undergoing AVF creation, to tailor management to individual cardiovascular profiles and improve overall clinical outcomes.

## Materials and Methods

This single-center, retrospective cohort study was conducted by the Section of Vascular Surgery, Department of Surgery, at The Aga Khan University Hospital, Karachi, Pakistan, one of the largest and most well-established tertiary care centers in the country. Data were collected from Health Information Management Services for all patients who underwent AVF creation between January 2014 and January 2022.

Adult ESRD patients with echocardiograms performed within 3 months before and 6 months after AVF creation were included. Patients with sepsis, a history of renal transplant or congenital cardiac defects, and significant acquired cardiac abnormalities (e.g., more than moderate valvular regurgitations) were excluded from the study. All echocardiograms were performed and reported by board-certified cardiologists using uniform institutional protocols. Measurements were abstracted from finalized reports. Because of the retrospective design, interobserver variability and blinding could not be independently assessed.

Patients were identified using the International Classification of Diseases, Ninth Revision (ICD-9) coding system. Medical records of all eligible patients were reviewed, and demographic, clinical, and procedural details were collected on a predefined and approved data collection pro forma.

Data were analyzed using Stata 15 software (StataCorp, College Station, TX, USA). Quantitative variables (weight, height, follow-up time, cardiac measurements) are presented as means with standard deviations or medians with interquartile ranges (IQR), while all categorical variables are presented as frequencies and percentages. A paired t-test was used to compare the means of preoperative and postoperative quantitative variables. Pre- and post-AVF creation Left ventricular ejection fraction (LVEF) were then compared. The Stuart–Maxwell test, a chi-squared test designed for paired and polytomous variables, was used to evaluate whether the distribution of LVEF significantly differed post-procedure. Survival analysis was conducted to analyze the time until MACE occurred. The Cox proportional hazards model was used to assess the effect of comorbidities (including atrial fibrillation, heart failure, hypertension, ischemic heart disease [IHD], and diabetes) on the occurrence of MACE, and hazard ratios (HR) were reported. A p-value of less than 0.05 was deemed statistically significant. A p-value of 0.25 was used for screening variables to be included in the multivariable model.

This study was conducted in accordance with the principles of the Declaration of Helsinki and followed the International Committee of Medical Journal Editors (ICMJE) recommendations for the conduct and reporting of research involving human subjects. Ethical approval was obtained via waiver from the Ethical Review Committee at Aga Khan University (AKU), Karachi, Pakistan (Ref: 2023-9307-26875; November 2, 2023). As this was a retrospective study, informed consent was waived. There was no direct interaction with participants. All patient data were deidentified and stored securely on password-protected computers accessible only to the research team.

### Operational definitions

ESRD: Stage 5 chronic kidney disease, with a glomerular filtration rate (GFR) of less than 15 mL/min.^11)^

MACE: the composite of total death, myocardial infarction, stroke, hospitalization because of heart failure, and revascularization, including percutaneous coronary intervention, and coronary artery bypass graft.^12)^

LVEF: The ratio fraction of chamber volume ejected in systole (stroke volume) in relation to the volume of the blood in the ventricle at the end of diastole (end-diastolic volume) and classified in line with American College of Cardiology guidelines: normal (>50%), mild dysfunction (40%–49%), moderate dysfunction (30%–39%) and severe dysfunction (<30%).^13)^

LVEF change: Transition between LVEF categories (normal, mild dysfunction, moderate dysfunction, and severe dysfunction).^13)^ Improvement was defined as a post-operative shift to a more favorable category (e.g., moderate to mild), while decline indicated a shift toward a higher degree of dysfunction (e.g., normal to mild).^14)^ Patients who remained within their baseline preoperative category post-procedure were classified as stable.^14)^

Low hemoglobin: Defined according to AKU guidelines as a serum hemoglobin level <12.3g/dL in males and <11.0g/dL in females.

## Results

A total of 119 patient records were included in the study. Patients had a mean age of 58.2 ± 13.6 years and body mass index of 27.0 ± 4.4, of which 66 (55.5%) patients were male. Existing comorbidities included heart failure in 34 patients (28.6%), diabetes mellitus in 84 patients (70.6%), hypertension in 114 patients (95.8%), atrial fibrillation in 5 patients (4.2%), and IHD in 69 patients (58.0%). Preoperative HD (35.3%) was catheter-based with no prior functioning AVFs or grafts, ensuring baseline measurements reflected physiological states without surgical shunts. Following the procedure, 2 patients (1.7%) with no prior history of cardiac illness were found to have developed new-onset mitral regurgitation. The types of AVF formed were basilic transposition in 46 patients (38.7%), brachiocephalic in 41 patients (34.5%), radiocephalic in 18 patients (15.1%), and lower limb AVF in 1 patient (0.8%). Arteriovenous bridge grafts were formed in 13 patients (10.9%). The median follow-up time was 14 months (IQR: 3–34). AVF was ligated in only 2 patients (1.7%) who developed anastomotic leak postoperatively (**Supplementary Table 1**). None of these patients had any major cardiac disease. Further demographic data are reported in **Supplementary Table 1**.

Comparison of echocardiographic parameters before and after AVF creation (results are summarized in **Supplementary Table 2**) showed a statistically significant increase in mean left atrial volume index (LAVI) (34.339 ± 10.361 vs. 37.964 ± 9.739, p = 0.001), as well as significant decreases in mean LVEF (50.798 ± 11.442 vs. 47.374 ± 12.277, p <0.001) and E′ velocity (0.052 ± 0.018 vs. 0.047 ± 0.016, p = 0.011). E′ velocity, representing early diastolic mitral annular tissue motion, reflects the rate of left ventricular (LV) relaxation; reduced values indicate impaired diastolic function and elevated filling pressures.^15)^ None of the other echocardiographic parameters showed significant differences. Results of the Stuart–Maxwell test for pre- and post-AVF LVEF categories (shown in **Supplementary Table 3**) showed a statistically significant change in the distribution of LVEF categories following AVF creation (χ^2^ = 8.390, p = 0.040, degrees of freedom = 3). Out of the 119 patients, 81 (68.07%) maintained a stable LVEF, 12 (10.08%) showed improvement and 26 (21.85%) experienced a decline.

Fifty-one patients (42.9%) experienced postoperative MACE, the majority of which included patients with non-ST-elevation myocardial infarction (18.5%). The median time to MACE after AVF creation was 8 months (IQR: 2–24). Stenosis was the most common AVF-related postoperative complication (21.0%). Cox proportional hazards regression (**Supplementary Table 4**) showed factors associated with the risk of MACE in patients with AVF. After adjustment, atrial fibrillation was associated with a significantly increased risk of MACE (HR: 7.420; 95% confidence interval [CI]:1.389–39.651; p = 0.019), as was preoperative severe LV dysfunction (HR: 3.210; 95% CI: 1.149–8.967; p = 0.026). This analysis, therefore, indicates that there is a significant association of atrial fibrillation and severe LV dysfunction with the occurrence of MACE. The statistical analysis of all the variables is reported in detail in **Supplementary Table 4**.

## Discussion

This study examined post-AVF echocardiographic changes to identify which parameters improved or declined following the procedure.

This cohort experienced a significant increase in mean LAVI post-AVF. LAVI has been shown to be one of the predictors of mortality and cardiovascular outcomes such as atrial fibrillation.^16,17)^ This reiterates the importance of considering this parameter when forming an AVF, especially in patients with preexisting atrial fibrillation. Increased LAVI has also been associated with other cardiovascular complications.^18)^ Patients with a history of cardiovascular events, therefore, might benefit from frequent cardiac monitoring, including regular ECGs, after this procedure considering the increased risk.

LV filling pressure is the ratio of early diastolic transmitral inflow velocity, known as E wave, to early diastolic mitral annular tissue velocity, known as E prime (E′) wave. The amplitude of these waves largely depends on the normal descent of the mitral annulus towards the apex during ventricular filling. A reduced mitral annular tissue velocity (E′) reflects impaired myocardial relaxation and is frequently observed in patients with diastolic dysfunction.^15)^ We observed a statistically significant reduction in the E′ wave in patients postoperatively, which can predict the impaired relaxation and compromised LV filling and can lead to signs and symptoms of heart failure with a normal LVEF. These indices can be used to complement other findings to establish the association.

Our results also indicated a significant decline in mean LVEF after the procedure. Existing literature on the impact of AVF creation on LVEF is mixed, with studies reporting stable, improved, or decreased function. Saka et al. observed a modest increase in LVEF (p = 0.038) among patients with baseline reduced or mildly reduced LVEF after dialysis access surgery.^14)^ Manzur-Pineda et al. reported an almost unchanged LVEF as a result of AVF creation.^19)^ However, Saleh et al. reported a decreased LVEF as a result of a high-flow AVF in HD patients.^20)^ In our cohort, a subset of patients with moderate and severe baseline LV dysfunction showed categorical improvement in LVEF following AVF creation. Although our study noted improved LVEF in select patients with preexisting moderate and severe LV dysfunction, this should not be interpreted as a universal protective effect. The paradoxical improvement in LVEF alongside a high MACE risk (HR: 3.210) in patients with severe baseline dysfunction likely reflects hemodynamic shifts rather than true myocardial recovery. AVF creation decreases afterload by reducing systemic vascular resistance and arterial stiffness, which can cause a numerical rise in LVEF without resolving the underlying pathology.^21)^ Furthermore, this systolic gain often masks a simultaneous decline in diastolic function, evidenced by significant increases in LAVI, which serves as a more reliable predictor of cardiac outcomes in ESRD patients.^15)^ Finally, selection bias may contribute, as clinicians typically reserve AVF creation for the most stable individuals within this high-risk cohort.^14)^ Therefore, LVEF improvement still necessitates vigilant risk stratification and monitoring.

Half of the patients with moderate LV dysfunction improved to either mild LV dysfunction (12.50%) or normal ventricular function (37.50%), and half of patients with severe ventricular dysfunction improved to either moderate LV dysfunction (33.33%) or mild LV dysfunction (16.67%) after AVF creation. We found that Saka et al. also reported improvement in select pre-AVF LVEF, albeit the improvements were in different LVEF categories compared to our study.^14)^ A decrease in arterial stiffness and blood pressure, leading to decreased afterload and an increased LVEF, may be a possible explanation.^21)^ It is worth noting the degree of LV dysfunction before proceeding with AVF creation, and since this finding is not uncommon, it should be studied further to determine its true significance given its potential to indicate better or worse outcomes.

Multivariable Cox proportional hazards model revealed that atrial fibrillation and preoperative severe LV dysfunction were significantly associated with increased occurrence of MACE in patients who underwent AVF creation. Atrial fibrillation’s occurrence is associated with an increased LAVI, and our study reports both an association of pre-AVF atrial fibrillation with post-AVF MACE and a post-AVF increase in mean LAVI.^16,17)^ It is recommended that pre-existing arrhythmias should be managed or corrected preoperatively.^22)^ Preoperative severe LV dysfunction was also significantly associated with the occurrence of MACE on multivariable analysis. However, a substantial portion of the existing literature associates this finding only with other major vascular surgeries. Patients undergoing major vascular surgery suffering from heart failure, among other comorbidities, are at an increased risk of experiencing perioperative MACE, but reported less than 1% risk in AVF patients.^22)^ Another study reported LVEF preoperatively to not be predictive of MACE, but excluded AVF.^23)^ Patients with heart failure or low LVEF are at greater MACE risk post-vascular access surgery due to reduced cardiac reserve, stress, arrhythmia, and fluid overload.^22)^ Although AVF is generally avoided in severe LV dysfunction, it is offered as long-term dialysis in select healthy, active patients in this setting, resulting in a few such cases (**Supplementary Table 3**). The contribution of AVF-related dysfunction versus pre-existing cardiac disease to MACE is unclear, warranting prospective studies.

While IHD returned a borderline p-value of 0.052 (adjusted HR: 2.004) in the multivariable analysis, it remains a critical clinical risk factor for postoperative MACE. The high prevalence of IHD (58.0%) in this cohort reflects the significant cardiovascular burden typical of South Asian ESRD patients, who frequently present with more advanced disease and at a significantly younger age than other populations.^2)^ Scientific literature confirms that preexisting IHD increases the risk of composite cardiac events in dialysis patients, as the heart’s reduced coronary reserve struggles to adapt to the striking physiological changes (specifically increased cardiac preload and output) triggered by the creation of an AVF.^8)^ Furthermore, chronic volume overload and uremic conditions in this population exacerbate the risk of myocardial ischemia and heart failure.^10)^ Therefore, preoperative IHD should be considered a major risk indicator, warranting stringent cardiovascular optimization and monitoring regardless of the narrow statistical threshold.^22)^

Our reported 42.9% incidence of MACE stands in contrast to the 15%–25% rates typically observed in HIC cohorts. This significant elevation in risk underscores an important clinical insight provided by our LMIC dataset: the extra work an AVF puts on the heart is more dangerous for patients who already have advanced disease when they first seek treatment. In LMIC cohorts like ours, where 95.8% of our patients were hypertensive and 70.6% were diabetic, the absence of early-stage renal and cardiac management (common in LMIC settings) likely results in a baseline cardiac environment that is far more vulnerable to the increased preload of a fistula than the more stabilized populations studied in HICs.^24)^ This proves that HIC-centric risk models are insufficient for predicting outcomes in the South Asian population.

These findings suggest several preliminary considerations for refining clinical decision-making in South Asian ESRD populations, though such strategies warrant further evaluation in larger prospective trials. Given that preoperative atrial fibrillation was associated with a 7.4-fold increase in MACE risk, it might be beneficial to prioritize the preoperative management or correction of arrhythmias to potentially mitigate adverse outcomes.^10)^ Furthermore, as severe LV dysfunction independently tripled the risk of MACE, implementing a highly individualized patient assessment (perhaps involving a multidisciplinary “Heart-Kidney” team review) could assist clinicians in weighing the hemodynamic stress of an AVF against a patient’s diminished cardiac reserve.^10)^ Regarding preoperative screening, it may prove efficacious to focus on echocardiographic indices such as the LAVI and E′ velocity because these parameters serve as sensitive markers of adverse structural remodeling and impaired myocardial relaxation, both of which showed significant deterioration post-AVF in this cohort. Finally, the establishment of a routine postoperative surveillance protocol, including follow-up echocardiography for high-risk individuals, might be beneficial for the early identification of structural decline. Such a proactive monitoring strategy could prove useful in addressing the 42.9% incidence of MACE by enabling more timely clinical or pharmacological adjustments.^25)^

This study adds to the limited literature evaluating echocardiographic changes following AVF creation in ESRD patients. However, there are some limitations of this study. This is a single-centered study, which may limit the generalizability of this study’s results. The cohort included a limited number of patients with moderate and severe LV dysfunction. Despite identifying a statistically significant change in the distribution of LVEF categories post-AVF creation using the Stuart–Maxwell test, the strength of our analysis would have increased with a larger sample size, and subgroup analysis would be possible. We recommend multicenter prospective studies with larger cohorts to validate and generalize these findings. Mean pulmonary artery pressures and AVF flow rates, which may influence cardiac function post-dialysis, could not be analyzed in this retrospective study due to unavailable data, necessitating a separate study to address their impact. Cause-specific mortality information was not consistently documented, limiting discrimination between cardiac and non-cardiac deaths; prospective studies should capture this distinction.

## Conclusions

AVF creation was associated with increased LAVI and decreased E′ velocity, suggesting adverse diastolic and structural changes. Severe preoperative LV dysfunction and atrial fibrillation were significantly associated with MACE, underscoring the need for extensive risk stratification, prior evaluation, and preemptive correction of arrhythmia. In select patients with moderate or severe LV dysfunction, we observed improvement in LVEF following AVF creation, suggesting a possible hemodynamic benefit in this subgroup. These findings emphasize the need for large-scale prospective studies to further investigate and study these associations, as well as the importance of individualized patient assessment when considering this procedure.

## Supplementary Materials

Supplementary Table 1 Demographic and clinical characteristics.

Supplementary Table 2 Echocardiography parameters.

Supplementary Table 3 Crosstabulation of ejection fraction categories pre-AVF and post-AVF.

Supplementary Table 4 Preoperative factors associated with the occurrence of MACE in patients with arteriovenous access.
