# Supplementary material for: Impact of Arteriovenous Access Formation on Cardiac Structure and Function in Hemodialysis Patients
Source: Ann Vasc Dis. 2026 Apr 23;19(1):25-00112. doi: 10.3400/avd.oa.25-00112 (PMC13110923; doi:10.3400/avd.oa.25-00112)
Supplement: Supplementary Table 1 — Demographic and clinical characteristics. [file avd-19-1-25-00112-s001.pdf]

Supplementary Table 1 Demographic and clinical characteristics (n=119)

**Characteristics**		**Frequency (%)**
**Age (mean ± SD)**		58.2±13.6
**Gender**	Male	66 (55.5)
	Female	53 (44.5)
**BMI (mean ± SD)**		27.0±4.4
**Comorbidities**	Existing heart failure	34 (28.6)
	Diabetes mellitus	84 (70.6)
	Hypertension	114 (95.8)
	Atrial fibrillation	5 (4.2)
	Ischemic heart disease	69 (58.0)
**Patients on hemodialysis**		42 (35.3)
**Type of arteriovenous access**	Radiocephalic	18 (15.1)
	Brachiocephalic	41 (34.5)
	Basilic transposition	46 (38.7)
	Arteriovenous bridge graft	13 (10.9)
	Lower limb	1 (0.8)
**Site of arteriovenous fistula (AVF)**	Left	82 (68.9)
	Right	37 (31.1)
**Blood parameters at time of arteriovenous fistula creation**	Hemoglobin (mean ± SD)	9.9±1.4
	Hematocrit (mean ± SD)	31.0±4.5
**Type of anesthesia**	Local	61 (51.3)
	General	50 (42.0)
	Block	7 (5.9)
	Monitored anesthesia care	1 (0.8)
**AVF-related complications**	Thrombosis/non-functional	8 (6.7)
	Stenosis	25 (21.0)
	Infection/hematoma/dehiscence	9 (6.7)
	Steal syndrome	1 (0.8)
	Anastomotic leak/rupture	2 (1.7)
**Follow-up of patients, in months (median, IQR)**		14 (3-34)
**Post-operative MACE**		51 (42.9)
**Gap between AVF creation and MACE, in months (median, IQR)**		8 (2-24)
**Post-operative cardiac complications**	Stable angina	4 (3.4)
	Unstable angina	11 (9.2)
	Non-ST elevation myocardial infarction	22 (18.5)
	ST elevation myocardial infarction	5 (4.2)
	Stroke	6 (5.0)
	Mitral valve regurgitation	2 (1.7)
**Cardiac complication management**	Coronary artery bypass grafting	35 (29.4)

BMI: body mass index; AVF: arteriovenous fistula; MACE: major adverse cardiac events; IQR: interquartile range

Supplementary Table 2 Echocardiography parameters (n=119)

**Echocardiography parameters**	**Pre-AVF creation**	**Post-AVF creation**	**p-Value**
	**(mean ± SD)**	**(mean ± SD)**	
LV internal dimension, end-diastole (mm)	42.56±6.13	42.31±7.32	0.740
LV internal dimension (mm)	27.90±6.96	28.38±8.25	0.539
LV septum (mm)	10.79±1.95	11.11±2.09	0.090
Posterior wall (mm)	10.14±1.76	10.34±1.96	0.293
LA volume index (ml/m²)	34.34±10.36	37.96±9.74	0.001[a]
Aortic root (mm)	28.16±3.14	27.88±3.53	0.410
LVOT diameter (cm)	1.85±0.19	1.85±0.18	0.736
LVOT VTI (cm)	19.89±5.67	19.94±4.56	0.949
Stroke volume of LVOT (ml)	54.56±17.23	55.55±16.59	0.716
Area 1 of LA (cm/sq)	19.86±3.96	21.91±13.81	0.154
Area 2 of LA (cm/sq)	19.49±3.51	20.03±3.34	0.207
Length 1 of LA (mm)	5.47±0.68	5.52±0.63	0.605
Length 2 of LA (mm)	5.28±0.61	5.24±0.70	0.674
LA volume (ml)	65.40±18.17	68.07±16.24	0.219
E velocity (m/s)	0.96±1.09	0.84±0.31	0.357
E' velocity (m/s)	0.052±0.018	0.047±0.016	0.011[a]
E/E' ratio	18.68±8.67	19.65±9.33	0.370
Ejection fraction	50.80±11.44	47.37±12.28	<0.001[a]

[a]Significant at <0.05 on paired t-test.

LV: left ventricular; LA: left atrial; LVOT: left ventricular outflow tract; VTI: velocity time integral; AVF: arteriovenous fistula

Supplementary Table 3 Crosstabulation of ejection fraction categories pre-AVF and post-AVF (n=119)

		LVEF post-AVF				Total
		Normal	Mild dysfunction	Moderate dysfunction	Severe dysfunction	
LVEF pre-AVF	Normal	66 (77.6%)	10 (11.8%)	7 (8.2%)	2 (2.4%)	85 (100.0%)
	Mild dysfunction	2 (14.3%)	7 (50.0%)	0 (0.0%)	5 (35.7%)	14 (100.0%)
	Moderate dysfunction	3 (37.5%)	1 (12.5%)	2 (25.0%)	2 (25.0%)	8 (100.0%)
	Severe dysfunction	0 (0.0%)	2 (16.7%)	4 (33.3%)	6 (50.0%)	12 (100.0%)
Total						119

	χ^2	p-Value	df
Stuart-Maxwell test	8.390	0.040*	3

*Significant at p-value <0.05.

Green boxes: improved ejection fraction; yellow boxes: unchanged ejection fraction; red boxes: declined ejection fraction.

Percentages are reported according to within-row frequencies.

LVEF: left ventricular ejection fraction; AVF: arteriovenous fistula; df: degrees of freedom

Supplementary Table 4 Pre-operative factors associated with the occurrence of MACE in patients with arteriovenous access (n=119)

Factors	Unadjusted hazard ratio (95% CI)	p-Value	Adjusted hazard ratio (95% CI)	p-Value
**Atrial fibrillation**				
Yes	3.374 (0.789-14.433)	0.101[a]	7.420 (1.389-39.651)	0.019[b]
No	Reference		Reference	
**LV function**				
Severe dysfunction	2.994 (1.189-7.540)	0.020[a]	3.210 (1.149-8.967)	0.026[b]
Moderate dysfunction	0.425 (0.126-1.434)	0.168[a]	1.043 (0.253-4.304)	0.953
Mild dysfunction	0.620 (0.255-1.505)	0.291	1.028 (0.371-2.849)	0.958
Normal	Reference			
**Diabetes**				
Yes	1.674 (0.851-3.293)	0.136[a]	2.281 (0.975-5.337)	0.057
No	Reference		Reference	
**Ischemic heart disease**				
Yes	1.744 (0.949-3.205)	0.073[a]	2.004 (0.994-4.037)	0.052
No	Reference			
**Type of anesthesia**				
General	0.585 (0.325-1.051)	0.073[a]	1.529 (0.444-5.270)	0.501
Block	0.377 (0.112-1.268)	0.115[a]	0.757 (0.185-3.092)	0.698
MAC	N/A†			
Local	Reference			
**AV access type**				
Basilic transposition	0.337 (0.151-0.754)	0.008[a]	0.299 (0.071-1.250)	0.098
Brachiocephalic	0.698 (0.311-1.568)	0.384	0.782 (0.326-1.877)	0.582
AV bridge graft	0.407 (0.137-1.209)	0.106[a]	0.426 (0.099-1.841)	0.253
Lower limb	N/A[c]			
Radiocephalic	Reference			
**Heart failure**				
Yes	0.437 (0.060-3.213)	0.416		
No	Reference			
**Hypertension**				
Yes	1.240 (0.169-9.076)	0.833		
No	Reference			
**Gender**				
Male	1.982 (0.553-1.742)	0.950		
Female	Reference			
**Serum hemoglobin**				
Low	0.760 (0.350-1.652)	0.488		
Normal	Reference			

[a]Significant at p value <0.25 on univariate analysis.
[b]Significant at p value <0.05 on multivariable analysis.
[c]Singular record;unable to analyze.
MACE: major adverse cardiac events; LV: left ventricular; MAC: monitored anesthesia care; AV: arteriovenous; CI: confidence interval
